# SARS-CoV-2 Superspread in Fitness Center, Hong Kong, China, March 2021

**DOI:** 10.3201/eid2709.211177

**Published:** 2021-09

**Authors:** Linsey C. Marr

**Affiliations:** Virginia Polytechnic Institute and State University, Blacksburg, Virginia, USA

**Keywords:** air change rates, coronavirus disease, COVID-19, disease control strategies, disease transmission, gymnasiums, SARS-CoV-2, severe acute respiratory syndrome coronavirus 2

**To the Editors:** I read with interest the article by Chu et al. (*1*), which concluded that poor ventilation might have contributed to a severe acute respiratory syndrome coronavirus 2 (SARS-CoV-2) superspreading event at a fitness center in Hong Kong, China. As an example of SARS-CoV-2 not spreading in a converse environment, I report the absence of apparent transmission at a gym in Montgomery County, Virginia, USA, that emphasized ventilation as part of its coronavirus disease (COVID-19) precautions upon reopening in June 2020. The gym increased ventilation by opening 10 exterior doors and keeping them open even during cold or inclement weather. The gym also limited class sizes, stressed hygiene, and required ≥10 feet of distancing. Masks were not worn.

With the doors closed, the air change rate was estimated to be 0.07 air changes/hour, corresponding to a ventilation rate of 7.6 L/second/person (L/s/p) on the basis of an occupancy of 10 persons, below the 10 L/s/p minimum recommended by ASHRAE (American Society of Heating and Air-Conditioning Engineers) for health clubs (*2*). With the doors open, these values were estimated to be 2.4 air changes/hour and 240 L/s/p.

On September 24, 2020, an instructor at the gym developed upper respiratory symptoms and lost his sense of smell and taste. He was tested for SARS-CoV-2 infection and received a positive result on September 28, 2020. That day, the gym owner contacted 50 persons who had attended ≥1 of the instructor’s classes during September 21–25, 2020 to notify them of potential exposure. During subsequent follow-up, none of these 50 persons reported any COVID-19 symptoms, and 5 people who got tested received negative results . It is likely that increasing ventilation greatly mitigated the risk of transmission (*3*). Subsequently, the gym acquired a CO_2_ sensor and kept the CO_2_ level, an indicator of respiratory emissions, well below 600 ppm (*4*) by adjusting the number of open doors.

Appendix Additional details on intervention to prevent spread of SARS-CoV-2 in a gym in Virginia, USA.
