# Supplementary material for: SARS-CoV-2 Superspread in Fitness Center, Hong Kong, China, March 2021
Source: Emerg Infect Dis. 2021 Sep;27(9):2507. doi: 10.3201/eid2709.211177 (PMC8386779; doi:10.3201/eid2709.211177)
Supplement: Appendix — Additional details on intervention to prevent spread of SARS-CoV-2 in a gym in Virginia, USA. [file 21-1177-Techapp-s1.pdf]

Article DOI: https://doi.org/10.3201/eid2709.211177

SARS-CoV-2 Superspread in Fitness Center, Hong Kong, China, March 2021

Appendix

Ventilation calculations

The gym is a standalone building measuring 100 ft × 50 ft with an inclined, peaked ceiling 11.25 ft–19.75 ft high. The gym has 4 sets of double doors, 3 single doors, and 3 garage doors (Appendix Figure 1). The air change rate with the doors closed was estimated by the tracer gas concentration decay method when the gym was unoccupied, on the basis of measurements of CO_2. The ventilation rate was then calculated by multiplying the air change rate by the volume of the gym (3,593 m^3). The volume flow rate through the gym with the doors open was estimated conservatively on the basis of the area through which air was exchanged by cross-ventilation through 4 single doors (total opening area 7.8 m^2) on one side of the building and additional doors on the opposite side of the building, with a wind speed of 0.3 m/s, corresponding to the lowest average daily wind speed during January–May 2020. The tracer gas decay method could not be used to estimate the air change rate with the doors open because the building could not be left unoccupied with the doors open for the required duration of $\geq$2 h. The direction of flow through the gym varied with the direction of the wind.

Timeline of events

On September 24, 2020, an instructor at the gym developed upper respiratory symptoms and lost his sense of smell and taste (Appendix Figure 2). On September 25, 2020, he was tested for severe acute respiratory syndrome coronavirus 2 (SARS-CoV-2 infection) by real-time reverse transcription PCR and received a positive result on September 28, 2020. He reported contact prior to illness onset with an acquaintance, who along with some of her roommates had also recently tested positive. During September 21–25, 2020, the instructor had led eighteen 1-hr classes potentially exposing a total of 50 class participants. At the beginning of each class, the

trainer spent several minutes explaining the workout and then led the class through various exercises, voicing instructions loudly and walking throughout the space. The day the trainer received his positive test result, the gym owner contacted the 50 class participants to notify them of their potential exposure. The health department communicated with the gym owner 2 d later, on September 30, 2020, and did not initiate tracing because those exposed did not meet the definition of close contacts ($\leq$6 ft for $\geq$15 min). During subsequent follow-up by the gym owner for 14 d after the last exposure, none of these 50 persons reported any coronavirus disease (COVID-19) symptoms. Five elected to get tested for SARS-CoV-2 $\leq$7 d of notification and tested negative by real-time reverse transcription PCR.

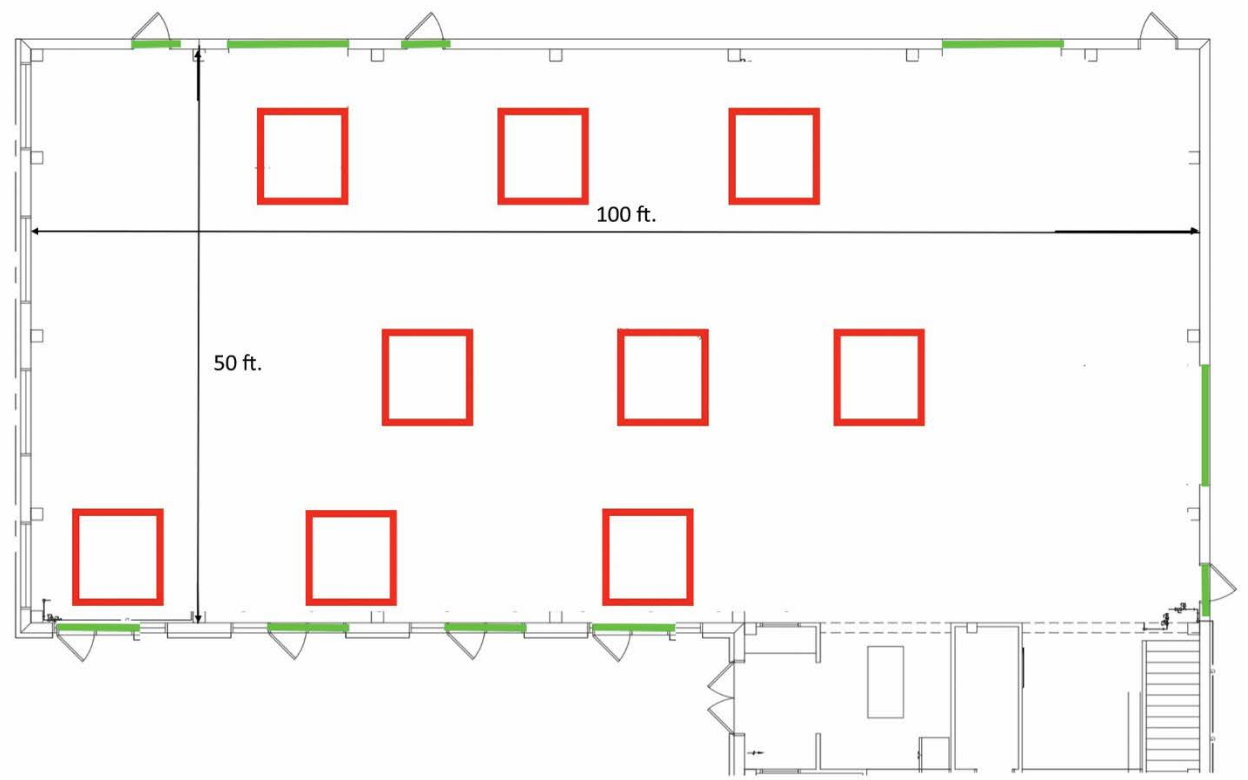

Appendix Figure 1. Diagram of interior of gym space that was part of investigation. Green lines indicate conventional and overhead garage doors that were at least partially open; red boxes indicate athlete workout stations.

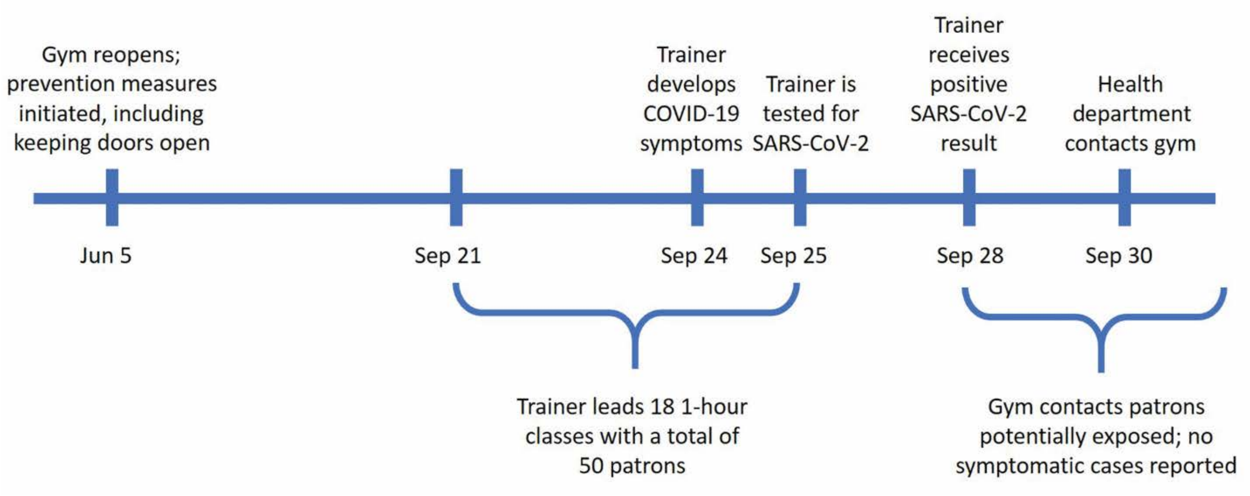

Appendix Figure 2. Timeline of events related to investigation of potential transmission of SARS-CoV-2 at a gym, Montgomery County, Virginia, USA, September 2020.
